# Mobile Network Coverage Prediction Using Multi-Modal Model Based on Deep Neural Networks and Semantic Segmentation

**DOI:** 10.3390/s24165178

**Published:** 2024-08-10

**Authors:** Sheng Zeng, Yuhang Ji, Weiwei Chen, Liping Yan, Xiang Zhao

**Affiliations:** College of Electronics and Information Engineering, Sichuan University, Chengdu 610065, China; zsxxyy@sina.com (S.Z.); jiyuhang@stu.scu.edu.cn (Y.J.); chenw564468275@163.com (W.C.); liping_yan@scu.edu.cn (L.Y.)

**Keywords:** coverage prediction, DNN, multi-modal model, satellite map, sematic segmentation

## Abstract

A coverage prediction model helps network operators find coverage gaps, plan base station locations, evaluate quality of service, and build radio maps for spectrum sharing, interference management, localization, etc. Existing coverage prediction models rely on the height and transmission power of the base station, or the assistance of a path loss model. All of these increase the complexity of large-scale coverage predictions. In this paper, we propose a multi-modal model, DNN-SS, which combines a DNN (deep neural network) and SS (semantic segmentation) to perform coverage prediction for mobile networks. Firstly, DNN-SS filters the samples with a geospatial-temporal moving average filter algorithm, and then uses a DNN to extract numerical features. Secondly, a pre-trained model is used to perform semantic segmentation of satellite images of the measurement area. Thirdly, a DNN is used to extract features from the results after semantic segmentation to form environmental features. Finally, the prediction model is trained on the dataset consisting of numerical features and environmental features. The experimental results on campus show that for random location prediction, the model achieves a RMSE (Root Mean Square Error) of 1.97 dB and a MAE (Mean Absolute Error) of 1.41 dB, which is an improvement of 10.86% and 10.2%, respectively, compared with existing models. For the prediction of a test area, the RMSE and MAE of the model are 4.32 dB and 3.45 dB, respectively, and the RMSE is only 0.22 dB lower than that of existing models. However, the DNN-SS model does not need the height, transmission power, and antenna gain of the base station, or a path loss model, which makes it more suitable for large-scale coverage prediction.

## 1. Introduction

Mobile network coverage prediction is the estimation of signal quality within a coverage area of an operator’s network. RSRP (Reference Signal Receiving Power) is used as a reference of communication quality and an indicator of network coverage in LTE networks. Coverage prediction models play an important role in network planning and deployment [1]. They can be used to assess signal quality before the actual deployment of a solution [2], and to identify coverage gaps in existing systems. These can help operators improve signal quality to enhance customer experience [3]. A coverage prediction model can also help build radio environment maps that can be used for spectrum sharing, interference management, localization, etc. [4,5,6]. Therefore, accurate coverage prediction is crucial for mobile network systems.

Over the years, many models were developed for coverage prediction. Although a path loss model such as COST-231-Walfisch–Ikegami has a simple structure and low computational complexity, the empirical formulas are inaccurate. Also, by only considering simple environmental types such as urban or suburban, they do not perform well in areas with different environmental characteristics [7]. The ray tracing model needs to rely on accurate 3D maps and materials to calculate the effects of absorption, scattering, refractions, and reflections on signal propagation, so the cost of 3D modeling and high computational complexity limits its application [8]. A prediction of interpolation method, such as Kriging, has good performance for locations near measurement points but poor performance for areas with no or only a few measurement points [9].

With very powerful learning and inferring capabilities, machine learning and deep learning are very efficient at mining information from high-dimensional data, which can significantly expedite data processing [10,11,12]. Researchers have started using machine learning and deep learning for coverage prediction and channel prediction [13,14,15,16,17,18,19,20,21,22,23]. In [13], the authors show the performances of four different machine learning models for predicting power values in urban environments. In [14], the authors utilize the machine learning method of extremely randomized tree to predict the RSRP and RSSI based on latitude and longitude without considering the effect of the environment. In [15], the authors develop a path loss model based on XGBoost. This model uses a CNN to extract image features from satellite maps. In [16], the authors use a multilayer perceptron (MLP) to predict path loss, considering only three environmental types to describe the propagation environment. In [17], the authors proposed a path loss model based on deep learning, which uses a path profile from 3D maps to represent the environment. In [18], the authors used a 3D electronic map of urban and RT simulations to help build a multitask learning model for channel characteristics. In [19], the authors proposed a coverage prediction model called FadeNet. The model uses building heights and topography as features, and processes satellite images using a CNN. In [20], the authors proposed a method based on deep learning that combines transmission power, antenna height, and satellite images to predict path loss, and uses VGG16 to extract environmental features from satellite images. In [21], the authors proposed a multi-modal channel prediction model using satellite maps and DeepChannel, with a RMSE and MAE of 2.6 dB and 2.06 dB, respectively. DeepChannel needs the height of base stations and the path loss model HataOkumara, and uses AlexNet to extract environmental features from satellite maps. In [22], the authors proposed an improved model called SS_DeepChannel for DeepChannel, where the model uses UNet, which is a semantic segmentation network, to obtain the environment type from satellite images, and then uses a CNN to extract environment features from images after semantic segmentation. The RMSE and MAE of the model are 2.21 dB and 1.57 dB, respectively, which is a significant improvement over DeepChannel. However, similar to DeepChannel, the model needs the height of base stations and the path loss model HataOkumara. In [23], the authors proposed a multi-modal model called Model-Aided DL that extracted features from satellite maps using CNNs. The RMSE of this model is 4.3 dB (811 MHz) and 4.10 dB (2630 MHz) in the test area. Model-Aided DL also needs the height and transmission power of base stations and the path loss models UMa_A and UMa_B, and extracts features from satellite images using a customized CNN.

These existing works have shown good performance. However, there are three problems as follows:

1. Much of the work relies on additional information about the base station, such as height, transmission power, antenna gain, etc. Some of the work also needs the assistance of a path loss model. Both increase the complexity for coverage prediction on a large scale.
2. Data augmentation such as translation and rotation are often needed to process satellite images. ResNet, VGG16, or a custom CNN are used to extract features from these images. These processes increase the complexity of the model.
3. Most existing works only discuss the results of the model on the test set. Since the test set comes from random sampling of the dataset, the prediction of the test set is essentially close to interpolation based on neighboring positions, without considering the prediction effect of the model in an unknown area without measurement points.

In this paper, we propose a model called DNN-SS for coverage prediction based on deep neural networks and semantic segmentation. The model relies only on the latitude and longitude of the base station and satellite maps. It decreases the conditions of coverage prediction and improves prediction accuracy.

The contributions of this work are composed as follows:

1. We designed the multi-modal model DNN-SS based on a DNN and semantic segmentation. The model does not rely on a path loss model or the height and transmission power of the base station, and only uses the latitude and longitude of a smartphone and base station combined with a satellite map of the measurement area to realize mobile network coverage prediction.
2. We used a pre-trained semantic segmentation model based on OCRNet (Object-Contextual Representations for Semantic Segmentation) to process satellite images of the measurement area. Then, we used a DNN to extract the rich environmental features of each measurement point from the results after semantic segmentation, which improved prediction performance.
3. We analyzed the possible fluctuation of data in large-scale measurements, and proposed a geospatial-temporal moving average filter algorithm to reduce the impact of outliers on the model.
4. Unlike existing works that focus on random location prediction, this paper discusses two cases to evaluate the generalization ability of the model: 1. random locations with some measurement data; and 2. a test area without measurement data. The measurement experiments on campus show that DNN-SS is validated for two cases, a random location and a test area, respectively, which demonstrates good performance of the proposed method for mobile network coverage prediction.

## 2. Measurement and Dataset

### 2.1. Environment of Measurement

**Figure 1:**
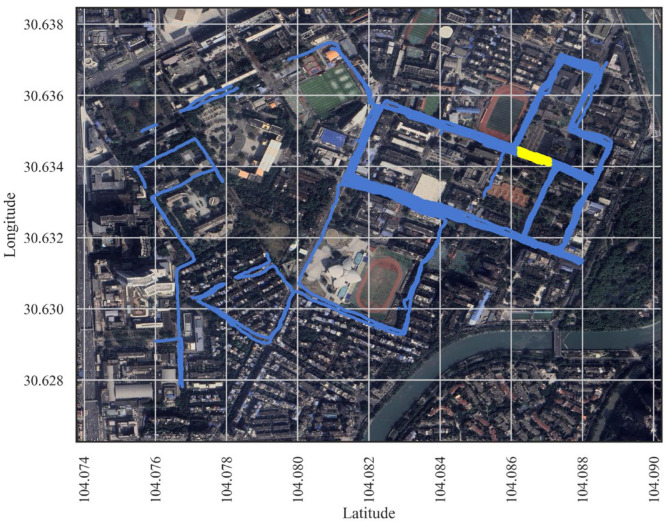
Satellite map of the experimental area. Blue indicates measurement points, with denser blue indicating more measurement points. The yellow area served as validation data for the test area case.

We designed a measurement experiment to verify the proposed method in the Wangjiang Campus of Sichuan University. In this work, measurements were conducted along the main roads within the campus to collect data. Figure 1 shows a satellite map of the experimental area. The whole experimental area is about 1.37 km^2^, covered by 40 different CIDs (Cell Identities). The blue lines indicate the measurement points, and denser blue means more measurement points. In order to simulate the spatial unevenness of the data measured by crowdsourcing, we measured more points in some areas and fewer points in some areas, and some areas were not involved in the measurements. The yellow area in the figure is our test area (about 110 m in length). This part was used to assess the performance of our model in unknown areas without measurement points.

### 2.2. Measurement Setup

We have designed a measurement system for collecting RSRP via a smartphone. The system consists of an Android-based measurement APP and a cloud-based data storage service. By calling the Android API, the measurement APP periodically records the timestamp, latitude and longitude, Earfcn (E-UTRA Absolute Radio Frequency Channel Number), TAC (Tracking Area Code), and CID (Cell Identity), and uploads them to a cloud server. The data storage service saves the data as a file in order to perform post-processing analysis. The details of the measurement system are in [10].

A researcher carried a smartphone with the measurement APP and walked at a constant speed on the main road in the experimental area of the campus. The measurement APP collected information such as the RSRP in the environment about once per second and uploaded it to a cloud server.

### 2.3. Satellite Maps

Satellite maps show the topographical characteristics of an area, including different buildings, plants, and even street-level details. This work uses satellite maps provided by Google. QGIS is a free and open-source geographic information system [24]. We used it to visualize Google satellite maps and obtain satellite images within the latitude and longitude of the experimental area using the Windows screenshot tool. The latitude and longitude of the satellite maps can be found in Figure 1. To reduce the cost of data storage and processing, our proposed method needs only one image of the experimental area instead of one satellite image for each measurement point.

### 2.4. Dataset

A dataset is defined as $D=\{S_{1},S_{2},\ldots ,S_{N}\}$, where N is the number of samples; $S_{1}$ denotes a sample in the dataset. $S_{i}=(f_{i},t_{i})$; *i* ∈ {1, 2, …, N}, $f_{i}$ refers to features, including timestamp, latitude, longitude, CID, and TAC; and $t_{i}$ is the label, which is the RSRP. We collected a total of 16,504 samples, each containing information such as measurement time, latitude and longitude of the measurement location, RSRP, and CID and TAC of the base station. Based on the CID and TAC of the base station, we could obtain the latitude and longitude of the base stations in the measurement area. We downloaded one satellite image of the experimental area of 3660 × 3154 pixels from Google as environmental data.

## 3. Methodology

### 3.1. System Architecture

**Figure 2:**
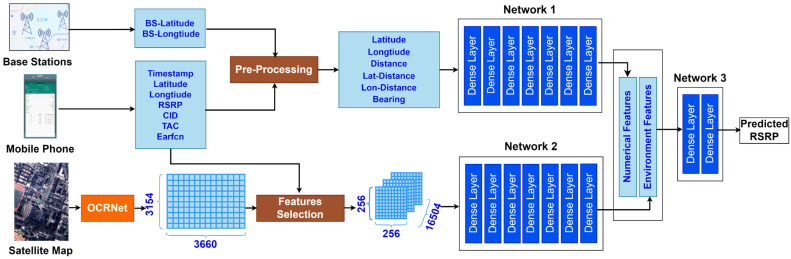
The architecture of DNN-SS.

Figure 2 shows the system architecture of the coverage prediction model DNN-SS based on a deep neural network and semantic segmentation. The light blue in Figure 2 represents the data, and the rectangular boxes in other colors represent the processing logic. DNN-SS consists of numerical feature extraction, environmental feature extraction, and a prediction network. Numerical feature extraction obtained the latitude, longitude, RSRP, and other data of the measurement point from the smartphone. With the Pre-Processing module, the data were filtered with a geospatial-temporal filter algorithm and combined with the coordinates of the base station to generate features such as distance, azimuth, etc. Then, Network 1 performed feature extraction to form the numerical features. Environmental feature extraction used semantic segmentation techniques based on OCRNet to process the satellite image of the experimental area, obtaining the environment type of each pixel and generating a 3660 × 3154 environment matrix. Then, with the Features Selection module, according to the latitude and longitude of the measurement point, the environment matrix was sliced into a 256 × 256 environment sub-matrix of each measurement point, and finally, feature extraction was performed by Network 2 to form the environmental features. Network 3 trained the data consisting of numerical features and environmental features to obtain the prediction of the RSRP. Details such as the parameters of the network are described in Section 4.1.

### 3.2. Numerical Features

#### 3.2.1. Geospatial-Temporal Moving Average Filter

**Figure 3:**
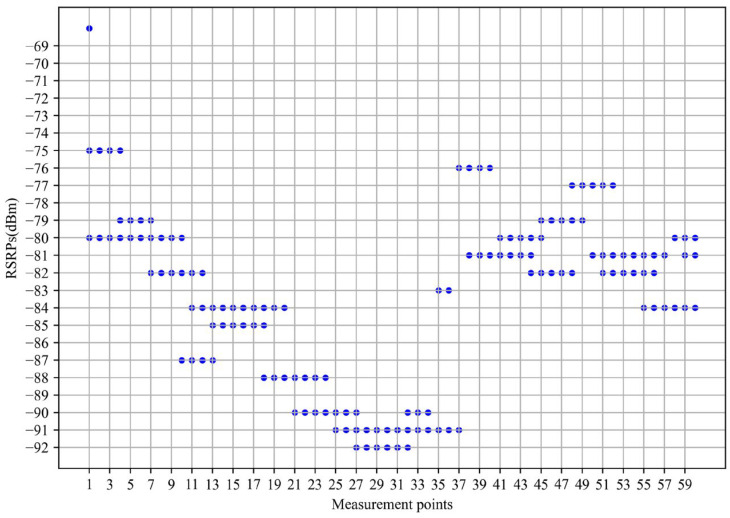
The fluctuation of the RSRPs around the measurement points (60 measurement points).

**Figure 4:**
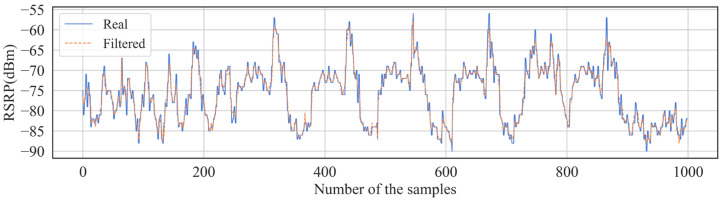
The effect of the geospatial-temporal moving average filter (1000 samples).

RSRPs measured in a short period at similar locations fluctuate largely due to multipath transmission and the fading effects of radio waves. Figure 3 shows fluctuations in the RSRP of 60 measurement points, with the horizontal axis showing the measurement point, and the vertical axis showing multiple RSRPs collected in 10 s within a 3 m radius of this measurement point. From the figure, for some measurement points, RSRPs measured over a short period of time have a maximum difference of about 10 dB. Therefore, we designed a geospatial-temporal moving average filter algorithm to process the RSRPs, which can reduce the impact of outliers on the model. The idea was to average the RSRPs collected over a short period around each measurement point to form the value of that measurement point. Algorithm 1 shows the logic of the algorithm. In our experiment, the distance in the algorithm was set to 3 m and the interval was set to 10 s. Figure 4 shows the effect of the geospatial-temporal moving average filter (1000 samples).
**Algorithm 1.** Geospatial-temporal moving average filter**Inputs:**Dataset D_raw_, Distance, Interval**Output:**Dataset D_new_1D_new_ ← {};2**for** item ∈ D_raw_
**do**3   group ← {item};4   **for** d ∈ D_raw_
**do**5       **if** (item.distance – d.distance) ≤ Distance **and**       (item.timstamp – d.timstamp) ≤ Interval **then**6       group ← group ∪ {*d*};7   item.RSRP = Mean (group.RSRP)8   D_new_ ← D_new_ ∪ {item};9**return** D_new_

#### 3.2.2. Handcrafted Features

Radio wave propagation is affected by the distance and azimuth between the base station and the UE (User Equipment) [25]. Therefore, we collected the latitude and longitude of all the base stations in the experimental area, and designed the distance from each measurement point to the base station, the distance in longitude, the distance in latitude, and the azimuth from the measurement point to the base station as features. In this paper, we use the Geodesic.WGS84.Inverse function from the GeographicLib library (version 2.0) to calculate the distance and azimuth between the measurement point and the base station [26].

### 3.3. Environmental Features

#### 3.3.1. Semantic Segmentation

Radio wave propagation is affected by the environment, and different environments cause different signal reflection, diffraction, attenuation, etc. Predicting the RSRP must consider the environment within a network coverage area [7,27]. Satellite maps provide some details of the environment. Therefore, we used a satellite map of the campus downloaded from Google Maps (QGIS 3.34) to show the environment of the experimental area. The map contained some of the streets around the campus and had a width of 3660 pixels and a height of 3154 pixels.

**Figure 5:**
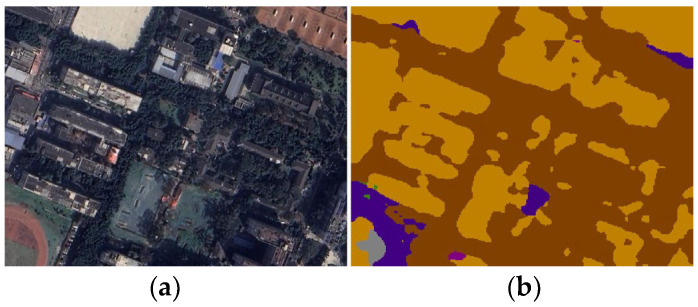
Semantic segmentation of the satellite image (one of the 16 sub-images): (**a**) satellite image. (**b**) The result of semantic segmentation by pre-training based on OCRNet.

Semantic segmentation refers to classifying the pixels in an image so that each pixel in the image belongs to a different class [28]. Some coverage prediction research has used semantic segmentation techniques to process satellite maps [22]. OCRNet (Object-Contextual Representations for Semantic Segmentation) is a network of semantic segmentation with good performance [29]. We divided the campus image of 3660 × 3154 pixels into 16 sub-images and then processed these 16 images using a pre-trained OCRNet model. We used a pre-trained model of OCRNet trained on a DLRSD dataset for semantic segmentation of satellite maps. The pre-trained model can recognize a total of 17 environment types: airplane, bare soil, buildings, cars, chaparral, court, dock, field, grass, mobile home, pavement, sand, sea, ship, tanks, trees, and water [30]. Each pixel on the original image was given a type of environment. Compared with the pure image features based on a CNN, the environmental features formed by the semantic segmentation results are higher-level environmental descriptions, which help to reduce the impact of variation of the image on the recognition of environmental features and improve the generalization of the model. Figure 5 shows the result of semantic segmentation. Figure 5a is the satellite image and Figure 5b is a visualization of the semantic segmentation result.

#### 3.3.2. Generation of the Environment Matrix

Some of the works used a simple CNN to extract the features of images after semantic segmentation [22]. In this work, we propose to use deep neural networks instead of a simple CNN to extract richer environmental features and improve prediction performance.

Instead of saving the result of semantic segmentation as an image, our proposed method saved it as a 3660 × 3154 matrix called an environment matrix. Each element in the environment matrix represents the environment type of the corresponding pixel on the satellite map. Then, according to the latitude and longitude of a measurement point, we obtained a sub-matrix of 256 × 256 centered on the measurement point from the environment matrix as the environment sub-matrix of the measurement point. Finally, the environment sub-matrix of each measurement point was fed into Network 2, and the environment features of that measurement point were extracted through Network 2. Algorithm 2 shows how to generate the environment sub-matrix of each measurement point, where Earea denotes the 3660 × 3154 environment matrix; the size of this matrix is the same as the size of the image of the experimental area, and each element corresponds to a pixel in the image. D denotes the set of all measurement points, and box denotes the coordinates in the latitude and longitude of the experimental area. Epoint denotes the environment sub-matrix of the measurement point. Height and width denote the image’s height and width, respectively. Size denotes the size of the sub-matrix, which is set to 256 in this paper. E denotes the set of all environment sub-matrices.
**Algorithm 2.** Generate environment sub-matrix set**Inputs:**E_area_, D, box, height, width, size**Output:**E1E ← {};2**for** item ∈ D **do:**3  //get x and y coordinates of measurement point from latitude and longitude4  x, y = ConvertLocation2Index (item.latitude, item.longitude, width, height, box);5   //get a submatrix of measurement point from E_area_ according to x and y coordinates6  E_point_ = GetSubMatrixByIndex (E_area_, x, y, size)7  E ←E ∪ {E_point_};8**return** E

### 3.4. Prediction Network

Network 3 is a prediction network for DNN-SS, consisting of a two-layer fully connected network that can predict the RSRP based on the input features. After processing of the Numerical Features Extraction and Environment Features Extraction, numerical features and environmental features are obtained for each measurement point. These two features are combined together to become the feature of the dataset and are used as inputs for Network 3.

### 3.5. Evaluation Metrics

Similar to most similar studies, we used RMSE (Root Mean Square Error) and MAE (Mean Absolute Error) to evaluate model performance. The RMSE is the square root of the MSE, which is the same as the original data scale, and can evaluate model accuracy more intuitively. The MAE reflects the absolute difference between the average predicted value and the actual value. Smaller values of these two indicators indicate better model performance.

(1) $$RMSE=\sqrt{\frac{1}{n}\sum_{i=1}^{n}{\left(y_{i}-\hat{y_{i}}\right)}^{2}}$$

(2) $$MAE=\frac{1}{n}\sum_{i=1}^{n}\left|y_{i}-\hat{y_{i}}\right|$$

In the above equations, *n* denotes the number of samples in the dataset; $y_{i}$ denotes the true value of sample; and $\hat{y_{i}}$ denotes the predicted value of sample *i*.

## 4. Experimental Results

### 4.1. Training Setup

We used Pytorch (Version 1.7.0) to build three full-connected neural networks (FCNNs) corresponding to Figure 2. Network 1 and Network 2 are six-layer FCNNs. Network 1 is used to extract numerical features from measurement data collected by smartphone, and Network 2 is used to extract environmental features from semantic segmentation results. Network 3 is a two-layer FCNN used to predict the value of the RSRP. Table 1 shows the detailed parameters of Network 1, Network 2, and Network 3 in Figure 2. We also set hyperparameters including learning rate, batch size, etc. The detailed settings of hyperparameters can be found in Table 2. In this paper, we used the pre-trained OCRNet model for semantic segmentation of satellite images of the experimental area. Since a CNN is no longer used to process satellite images, image enhancement is removed, which simplifies the training process and reduces the complexity of training.

### 4.2. Prediction of Random Locations

We discuss the results of the coverage prediction model in two cases: 1. random locations and 2. a test area. Since the test set comes from random sampling of the dataset, the test set can be used for the random location prediction case. In this paper, 80% of the dataset is used as the training set and 20% as the test set, and the predictions for the test set are referred to as random location prediction to distinguish them from the prediction for the test area below.

**Figure 6:**
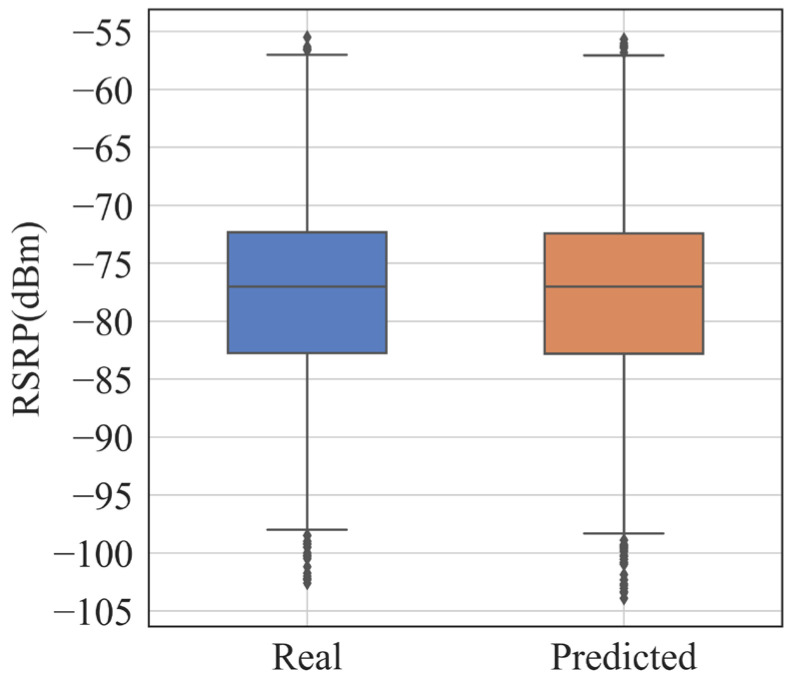
Box plot of real and predicted RSRPs at random locations.

**Figure 7:**
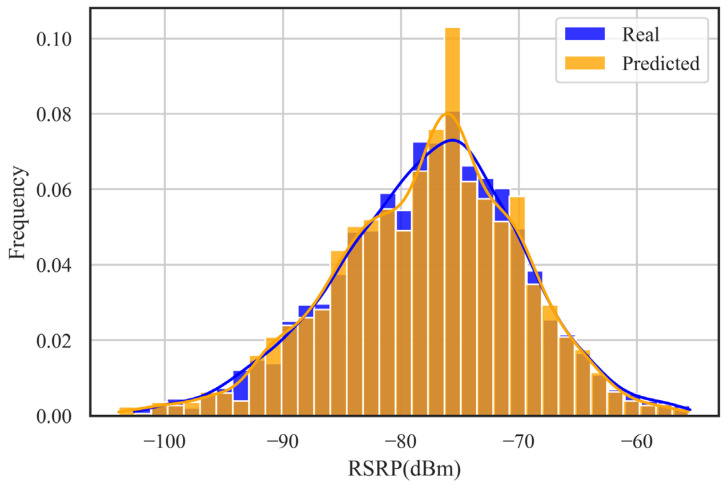
Histogram of real and predicted RSRPs at random locations.

**Figure 8:**
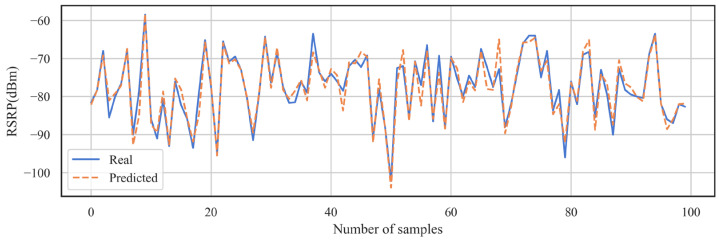
Comparison between real and predicted RSRPs (100 samples).

In this paper, the results of the DNN-SS model are evaluated by the RMSE and MAE. Table 3 shows the performance of the DNN-SS model on the training set and the test set. The RMSE and MAE of the model on the training set are 1.51 dB and 1.08 dB, respectively, and the RMSE and MAE of the model on the test set are 1.97 dB and 1.41 dB, respectively. Table 4 shows the statistical information of the predicted RSRP on random locations. From Table 4, it can be seen that the mean, median, and STD are very close between the real RSRP and the predicted RSRP, and the coefficient of variation shows the similarity between the two distributions. Figure 6 shows the box plot between the real data and the predicted data. Figure 7 shows the histogram of the real data versus the predicted data on the test set. From these two plots, it can be seen that the predicted results of DNN-SS are not much different from the real data, and the overall trend of the data is close. Figure 8 shows the case of 100 samples, and the predicted data are close to the real data, which can express the fluctuation trend of the real data well.

**Figure 9:**
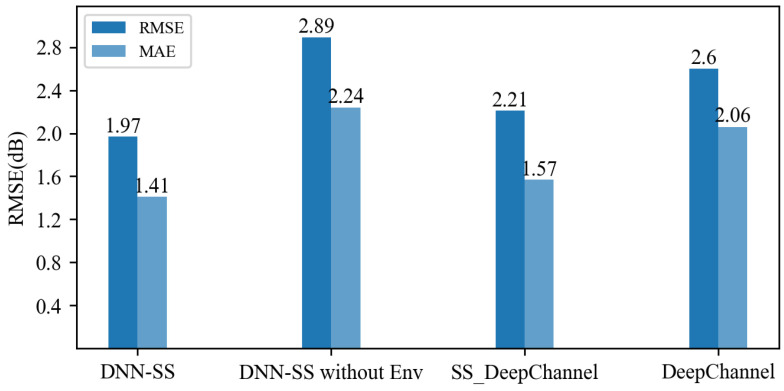
Comparison of RMSE and MAE at random locations.

Figure 9 compares the RMSE and MAE of the models SS_DeepChannel and DeepChannel to those of the DNN-SS model [21,22]. The former two models use deep learning combined with satellite images to achieve coverage prediction for random locations, where SS_DeepChannel is the best-performing model. From Figure 9, compared to those of the SS_DeepChannel model, the RMSE and MAE of our proposed DNN-SS model are improved by 10.86% and 10.2%, respectively. Compared to DeepChannel, the RMSE and MAE of our model are improved by 24.23% and 31.55%, respectively.

An ablation study was conducted to investigate the necessity of environmental feature extraction techniques. The DNN-SS model without an Env model, which uses only numerical features as a baseline model, was compared with the DNN-SS model that uses numerical and environmental features. Figure 9 also shows the results of the ablation study on the model. The RMSE of the base model (DNN-SS without Env model) is 2.89 dB, and the MAE is 2.24 dB, but the RMSE of the DNN-SS model is 1.97 dB, and the MAE is 1.41 dB. The contribution of environmental features to the DNN-SS model is 0.92 dB, while the contribution of environmental features of the MA-DL model in [23] is 0.8 dB, showing an improvement of 0.12 dB. This means high-level environmental features from semantic segmentation can effectively represent the real environment, which is useful for improving the generalizability of the model. In addition, it shows the full-connected network may be better than a simple CNN network in extracting environmental features. Meanwhile, data processing is simpler because there is no image augmentation.

### 4.3. Prediction of Test Area

Section 4.2 describes the prediction of random locations, and this section describes the prediction of the DNN-SS model for the test area. In the experimental area, an area was selected as the test area (Figure 1 yellow area); the data in it were used to evaluate model performance, and the data in the other areas were used as training data. The model was validated in the test area, which is referred to as prediction in the test area in this paper. Most of the current research work on coverage prediction rarely deals with prediction of the measurement region.

**Figure 10:**
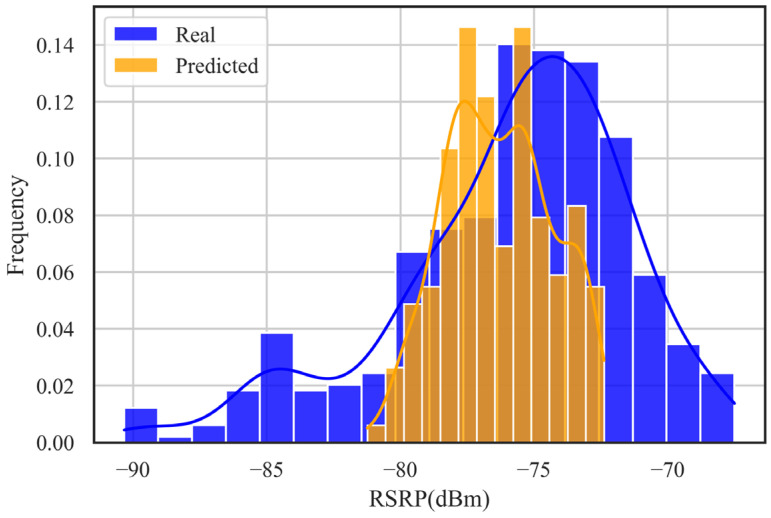
Histogram of real and predicted RSRPs at test area.

In this paper, we selected a 110 m road in the experimental area as the test area; the yellow area in Figure 1. The DNN-SS model predicts the RMSE and MAE for the test area, which are 4.32 dB and 3.45 dB, respectively. Table 5 shows the statistical information of the predicted data versus the real data in the test area. The mean, median, and STD of them differ by 0.49 dB, 1.3 dB, and 0.41 dB, respectively, with a large difference in the coefficient of variation. Figure 10 shows a histogram of the real data versus the predicted data within the test area. From the figure, there are two problems with the DNN-SS model; one is that the predicted range is relatively narrow and does not cover the whole range of the RSRP, and the other is that the center of the predicted histogram is slightly shifted to the left. This reflects that the trend of the predicted data is slightly different from that of the real data. This may be due to the complex environment of the experimental area, which did not measure data similar to the test area, limiting the performance of the model. Therefore, collecting data from more diverse environments through crowdsourcing has the potential to improve model performance.

**Figure 11:**
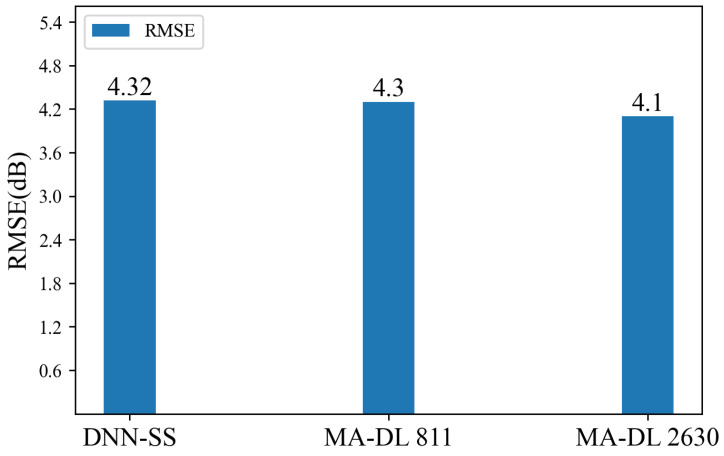
Comparison of RMSE and MAE at test area.

Figure 11 shows the RMSE metrics of the DNN-SS and MA-DL models in the test area. In [23], the MA-DL model is discussed in two frequency bands, 811 MHz and 2630 MHz, with a RMSE of 4.3 dB and 4.1 dB, respectively. The DNN-SS model is only 0.22 dB lower than the best-performing MA-DL 2630 and 0.02 dB lower than MA-DL 811.

### 4.4. Comparison of Models

Most of the existing research on coverage prediction relies on various information from the base station (such as height or antenna gain) or the assistance of a path loss model. Table 6 compares some of the works on coverage prediction using deep learning and satellite images. Table 6 shows the differences among the DNN-SS, SS_DeepChannel, DeepChannel, and Model-Aide DL models in terms of receiver parameters, base station parameters, satellite images, path loss model, and network type. The DNN-SS model needs only the latitude and longitude of the UE and the base station, as well as satellite images of the experimental area, and does not need the height of the base station or the aid of a path loss model; it is the least dependent among the four models.

## 5. Conclusions

In this paper, DNN-SS, which is a multi-modal model based on a DNN and semantic segmentation, is designed for coverage prediction in mobile networks. Compared to recent work, the DNN-SS model shows a large performance improvement in both the RMSE and MAE of random locations. For prediction of the RSRP on a test area without measurement points, the RMSE is slightly lower than that of existing work, which may be due to the complex experimental environment where it is difficult to obtain data similar to that of the test area, limiting the prediction capability of the model. Collecting data from more diverse environments through crowdsourcing has the potential to improve model performance. The DNN-SS model does not depend on the height or transmission power of the base station or the assistance of a path loss model, which reduces the dependence of coverage prediction. At the same time, it does not need one image for each measurement point or image augmentation, which decreases the cost of data storage and processing. In addition, for the fluctuation of smartphone measurement data, the DNN-SS model performs a geospatial-temporal moving average filter algorithm to better adapt to crowdsourced measurements. All these make the DNN-SS model more suitable for large-scale coverage prediction research. Since several signal parameters measured by smartphones are associated with electromagnetic radiation indicators, the model in this paper can also be expected to be applied to assess electromagnetic radiation.

## Figures and Tables

**Table 1:** Network architecture.

Network	Type	Input Size	Output Size	Neurons	Activation	Normalization	Dropout
Network 1	Dense Layer	6	200	200	ReLU	Batchnorm1d	
	Dense Layer	200	200	200	ReLU	Batchnorm1d	0.1
	Dense Layer	200	200	200	ReLU	Batchnorm1d	0.1
	Dense Layer	200	200	200	ReLU	Batchnorm1d	0.1
	Dense Layer	200	200	200	ReLU	Batchnorm1d	0.1
	Dense Layer	200	200	200	ReLU	Batchnorm1d	0.1
	Dense Layer	200	100	100	Linear		
Network 2	Dense Layer	256 × 256	200	200	ReLU	Batchnorm1d	
	Dense Layer	200	200	200	ReLU	Batchnorm1d	0.1
	Dense Layer	200	200	200	ReLU	Batchnorm1d	0.1
	Dense Layer	200	200	200	ReLU	Batchnorm1d	0.1
	Dense Layer	200	200	200	ReLU	Batchnorm1d	0.1
	Dense Layer	200	200	200	ReLU	Batchnorm1d	0.1
	Dense Layer	200	100	100	Linear		
Network 3	Desen Layer	100	16	16	ReLU	Batchnorm1d	
	Desen Layer	16	1	1	Linear		

**Table 2:** Hyperparameters for network.

Parameter	Value
Optimizer	Adam
Loss function	MSE
Learning rate	0.001
Weight decay	0.01
Dropout	0.1
Batch size	128
Epochs	30

**Table 3:** RMSE and MAE of training set and test set.

Dataset	RMSE (dB)	MAE (dB)
Training set	1.51	1.08
Test set	1.97	1.41

**Table 4:** Statistics of predicted and real RSRPs at random locations.

Parameter	Real RSRP	Predicted RSRP
Mean (dB)	−77.65	−77.74
Median (dB)	−77	−77.01
STD (dB)	7.94	7.82
Coefficient of variation	−10.22	−10.06

**Table 5:** Comparison of statistics between real and predicted RSRP at test area.

Parameter	Real RSRP	Predicted RSRP
Mean (dB)	−75.84	−76.33
Median (dB)	−75.20	−76.50
STD (dB)	4.36	3.95
Coefficient of variation	−5.76	−2.60

**Table 6:** Comparison of the dependence of the models.

Models	Receiver Parameters	Base Station Parameters	Satellite Images	Path Loss Model	Network Type
DNN-SS	Latitude, Longitude	Latitude, Longitude	Only one image for whole measurement area		DNN, OCRNet
SS_DeepChannel	Latitude, Longitude, Height of the receiver	Latitude, Longitude, Heights of the transmitter, operation frequency	One image per measurement point	HataOkumara	DNN, UNet, CNN
Model-Aided DL	Latitude, Longitude	Latitude, Longitude, Heights of the transmitter, Transmission power	One image per measurement point	UMa_A, UMa_B	DNN, CNN
DeepChannel	Latitude, Longitude, Height of the receiver	Latitude, Longitude, Heights of the transmitter, operation frequency	One image per measurement point	HataOkumara	DNN, CNN

## Data Availability

The data presented in this study are available on request from the corresponding author.
